# CAND1 is required for pollen viability in *Arabidopsis thaliana*—a test of the adaptive exchange hypothesis

**DOI:** 10.3389/fpls.2022.866086

**Published:** 2022-07-28

**Authors:** Lihong Li, Melaku Garsamo, Jing Yuan, Xiaojin Wang, Susan H. Lam, Kranthi Varala, Leonor C. Boavida, Yun Zhou, Xing Liu

**Affiliations:** ^1^Department of Biochemistry, Purdue University, West Lafayette, IN, United States; ^2^Center for Plant Biology, Purdue University, West Lafayette, IN, United States; ^3^Department of Botany and Plant Pathology, Purdue University, West Lafayette, IN, United States; ^4^Department of Horticulture and Landscape Architecture, Purdue University, West Lafayette, IN, United States

**Keywords:** reproductive development and death, adaptive response, Ubiquitin ligase (E3), CAND1, *Arabidopsis*, Male gametophyte

## Abstract

The dynamic assembly of SKP1•CUL1•F-box protein (SCF) ubiquitin ligases is important for protein ubiquitination and degradation. This process is enabled by CAND1, which exchanges F-box proteins associated with the common CUL1 scaffold, and thereby, recycles the limited CUL1 core and allows diverse F-box proteins to assemble active SCFs. Previous human cell biological and computational studies have led to the adaptive exchange hypothesis, which suggests that the CAND1-mediated exchange confers plasticity on the SCF system, allowing cells to tolerate large variations in F-box protein expression. Here, we tested this hypothesis using *Arabidopsis thaliana*, a multicellular organism expressing hundreds of F-box protein genes at variable levels in different tissues. The *cand1* null mutant in *Arabidopsis* is viable but produce almost no seeds. Bioinformatic, cell biological, and developmental analyses revealed that the low fertility in the *cand1* mutant is associated with cell death in pollen, where the net expression of F-box protein genes is significantly higher than any other *Arabidopsis* tissue. In addition, we show that the transmission efficiency of the *cand1* null allele was reduced through the male but not the female gametophyte. Our results suggest that CAND1 activity is essential in cells or tissues expressing high levels of F-box proteins. This finding is consistent with the proposed adaptive exchange hypothesis, demonstrating the necessity of the evolutionarily conserved CAND1-mediated exchange system in the development of a multicellular organism.

## Introduction

Majority of proteins in eukaryotic cells can be post-translationally modified at some point by the small protein ubiquitin (Ciechanover, 1998; Morreale and Walden, 2016). This process, referred to as ubiquitination, is one of the key mechanisms that allows for regulation of half-lives, activities, and localization of proteins (Pickart, 2001; Callis, 2014). Three types of enzymes (E1, E2, and E3) are sequentially employed for protein ubiquitination, the last of which, an E3 ligase, transfers ubiquitin to the target protein (Callis, 2014; Morreale and Walden, 2016). An important category of E3s are the cullin-RING ligases (CRLs), which are protein complexes characterized by a common cullin (CUL) backbone, the RING-containing protein RBX1 or RBX2, and a variety of interchangeable substrate receptor modules that recruit substrates for ubiquitination (Kelley, 2018; Rusnac and Zheng, 2020; Harper and Schulman, 2021). SKP1•CUL1•F-box protein (SCF) E3 ligases, the founding members of CRLs, comprise the CUL1•RBX1 core, the F-box protein substrate receptor, and the adaptor protein SKP1 that allows the assembly of F-box proteins with CUL1 (Rusnac and Zheng, 2020; Harper and Schulman, 2021). The modularity of the SCF complex enables SCFs to target diverse protein substrates, and the specific F-box protein assembled with CUL1 determines the substrate specificity (Hua and Vierstra, 2011; Lydeard et al., 2013). Different organisms contain different numbers of F-box protein genes—69 in human (Liu et al., 2020), ~520 in *C. elegans* (Thomas, 2006), and ~ 700 in *Arabidopsis thaliana* (Hua et al., 2011)—so potentially, dozens to hundreds of SCFs with distinct composition may be assembled in a single eukaryotic cell.

At the post-translational level, multiple factors cooperatively regulate the assembly and activity of SCF complexes. When the ubiquitin-like protein NEDD8 is conjugated to CUL1 through an ATP-dependent process termed as neddylation, it induces multiple protein–protein interactions within an SCF-E2 complex, converting the complex to a configuration that favors substrate ubiquitination (Baek et al., 2020, 2021; Horn-Ghetko et al., 2021). After the substrate is ubiquitinated, NEDD8 is subsequently cleaved from CUL1 by COP9 signalosome (CSN), which inactivates SCF and allows CUL1 to bind CAND1, the F-box protein exchange factor (Pierce et al., 2013; Liu et al., 2018). In the absence of CUL1 neddylation, CAND1 significantly accelerates the dissociation of an SCF but marginally affects the association rate of a new SCF. As a result, CAND1 recycles CUL1 from an SCF that has been deactivated by CSN, and then exchanges the F-box protein recruited to the CUL1 core. Upon assembly of the new SCF, CAND1 dissociates from CUL1, immediately after which CUL1 is conjugated by NEDD8, preventing the binding of CAND1 and locking the new SCF at a stable and activated state (Liu et al., 2018; Wang et al., 2020). With the combined regulatory effects from NEDD8, CSN, and CAND1, CUL1 constantly and quickly cycles between the active (neddylated SCF) and inactive (CAND1 bound) status. This cycling allows a limited pool of CUL1 to rapidly scan the entire population of F-box proteins, ensuring timely recruitment of substrate-bound F-box proteins and the ubiquitination of the substrate. Without the CAND1-mediated exchange of F-box proteins, SCFs remain stably assembled whether their activities are required or not, resulting in a static pool of SCFs that fail to ubiquitinate newly formed SCF substrates in time (Reitsma et al., 2017; Liu et al., 2018). This understanding of the CAND1 functional mechanism well explains the reduced SCF activities found in cells and organisms lacking the *CAND1* gene (Cheng et al., 2004, 2020; Chuang et al., 2004; Feng et al., 2004; Alonso-Peral et al., 2006; Lo and Hannink, 2006; Zhang et al., 2008; Bosu et al., 2010; Kim et al., 2010; Wang et al., 2011; Pierce et al., 2013; Wu et al., 2013; Zemla et al., 2013; Reitsma et al., 2017; Liu et al., 2018). The natural question following this conceptual insight is, why is this complex and energy-consuming exchange system advantageous to eukaryotes?

**Figure 1:**
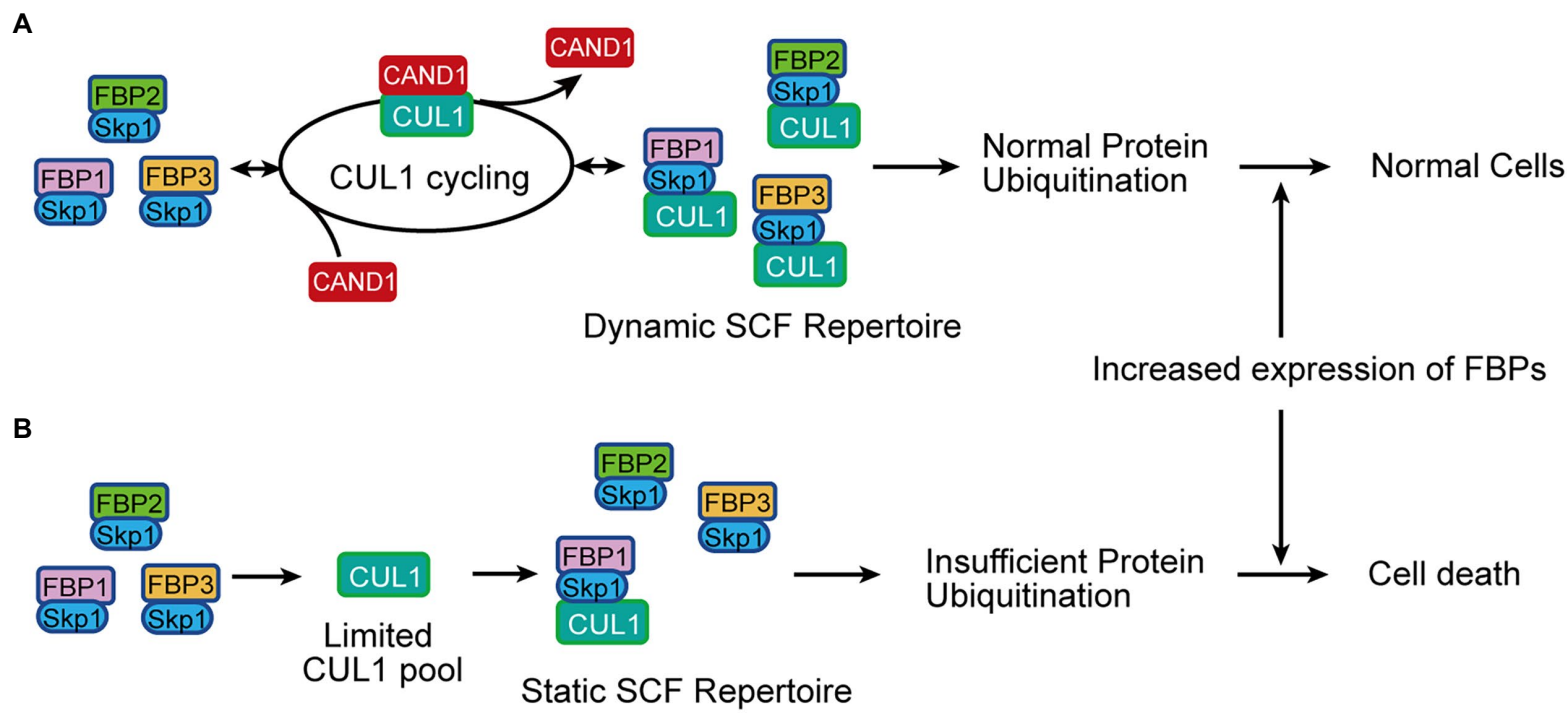
Schematic illustrating the adaptive exchange hypothesis to be tested in this study. **(A)** In the presence of the CAND1-mediated exchange, CUL1 is quickly recycled from an inactive SCF to assemble a new SCF. Thereby, the limited pool of CUL1 constantly scans F-box proteins available in the cell, forming a dynamic pool of SCFs that tolerates variations in the expression of F-box proteins and maintains normal protein ubiquitination in cells under different conditions. **(B)** In the absence of CAND1, the cellular pool of SCFs becomes static and the formation of new SCFs is slow and insufficient. This leads to defects in protein ubiquitination, and when levels of F-box protein expression increase significantly, the deficiency in protein ubiquitination becomes more severe and may eventually result in cell death. FBP in **(A,B)**: F-box protein.

To explain the advantage of the exchange system, an “adaptive exchange” hypothesis was proposed: the rapid exchange cycle allows cells to tolerate large variations in the expression of individual F-box proteins (Figure 1; Liu et al., 2018). Consistent with this hypothesis, computational simulation predicted that increasing the total cellular F-box protein concentration would stabilize an SCF substrate in the absence, but not in the presence, of CAND1 (Liu et al., 2018). Moreover, overexpressing an F-box protein gene in cultured human HEK293 cells led to cell death in *CAND1/2* knockout cells but no visible defects in the normal cells (Liu et al., 2018). This experimental result further supports the adaptive exchange hypothesis, and more importantly, it suggests that without the CAND1-mediated exchange, a multicellular organism may fail to maintain cells or organs that express high levels of F-box proteins. Thus, in this study, we aimed to test this possibility using a multicellular organism as the experimental model.

The genome of *A. thaliana*, a multicellular flowering plant, encodes one CUL1 protein, one CAND1 protein, and an exceptionally large number of F-box proteins (~700; Hua et al., 2011). Unlike *Cand1* knockout mice that are homozygous lethal at preweaning stages (record from International Mouse Phenotyping Consortium), the *Arabidopsis cand1* loss-of-function mutant is viable despite various SCF-related deficiencies and developmental defects (Cheng et al., 2004; Chuang et al., 2004; Feng et al., 2004; Alonso-Peral et al., 2006). Furthermore, it was reported that the regulation of SCF assembly and disassembly cycle by CAND1 is required for optimal SCF activity in *Arabidopsis* (Zhang et al., 2008). These features make *Arabidopsis* an ideal system to study the biological role of CAND1-mediated exchange. Here, we performed bioinformatic, cell biological, and developmental analyses on *Arabidopsis* wild-type and the *cand1* mutant to test our adaptive exchange hypothesis.

## Experimental procedures

### Bioinformatic analysis

To generate the heat map of gene expression levels, RNA-seq data were obtained from the Araport Database (Krishnakumar et al., 2015), which include values of Transcripts Per Million (TPM) for 468 F-box protein gene transcripts in eight *Arabidopsis* tissues (pollen, receptacle, root apical meristem, stage 12 inflorescence, leaf, shoot apical meristem, root, and carpel). A subset of 346 F-box genes that showed greater than 1 TPM in at least one tissue were retained. The heatmap displaying this data was generated using the ComplexHeatmap package (Gu et al., 2016) in R. Gene expression values were row-scaled for visualization and the rows were clustered by Euclidean distance. Further, the row cluster dendrogram was empirically split into 6 groups. A second heatmap of total TPM was added to display the variation in net expression levels of individual F-box genes. The bar graph showing total pools of F-box protein gene transcripts was generated by summing levels of the 346 F-box protein gene transcripts within a given tissue. To compare the net expression of F-box proteins in various tissues, quantitative and normalized gene expression data for 406 F-box protein genes across 135 samples (45 types of tissue, three replicates per tissue type) were downloaded from the ePlant database (Waese et al., 2017). These values were summed for each sample, averaged for the same tissue type, and plotted as a bar graph with standard errors.

### Plant materials and growth conditions

The wild-type, mutant, and transgenic plants of *A. thaliana* used in this study were in Columbia ecotype (Col-0) background. Seeds of the previously characterized T-DNA insertional mutant *cand1-3* (SALK_110969; Cheng et al., 2004; Feng et al., 2004; Alonso-Peral et al., 2006) were obtained from the Arabidopsis Biological Resource Center (Ohio State University, Columbus, OH, United States). All seeds were surface sterilized by incubation in 70% ethanol for 2 min, followed by 30% commercial bleach (to yield 1.8% sodium hypochlorite) for 5 min, and washed with sterile water multiple times. Seeds were sown on Murashige and Skoog (MS) medium containing 1% sucrose and 0.8% (w/v) agar, pH 5.7. Agar plates were incubated at 4°C for 48 h in the dark for stratification and then transferred to a growth room at 22°C with a long-day (16 h light/ 8 h dark) condition. After 10 days, seedlings were transferred to soil. Wild-type, heterozygous or homozygous *cand1-3* mutant plants were determined by extracting genomic DNA from leaves of individual plant (Edwards et al., 1991), followed by PCR using the T-DNA-specific primer (T: 5′-ATTTTGCCGATTTCGGAAC-3′) and two gene specific primers (F: 5′-TATGCTCTTGGAAACATTGCC-3′, and R1: 5′-CATCCACAACATGCTTGAATG-3′; Supplementary Figure S1A). The *pCAND1::H2B-GFP* transgenic plants were generated by introducing a pMoA34 construct (Barrell and Conner, 2006) into Col-0 *Arabidopsis* via *Agrobacterium*-mediated transformation (Hwang et al., 2017). The pMoA34 construct carries the following elements in the presented order: 3 kb genomic DNA sequence immediately upstream to *CAND1*, the DNA sequence encoding the H2B-GFP reporter, 1 kb genomic DNA sequence immediately downstream of *CAND1*. Multiple independent transgenic plants were obtained and used for confocal imaging analyses. The *cand1-3;pCAND1::CAND1^3xFLAG^* complementation transgenic plants were generated using similar experimental procedures to introduce a pMoA34 construct into *cand1-3*/+ heterozygous plants. The complementation construct carries a 12.9-kb genomic fragment including the 3 kb genomic DNA sequence immediately upstream to *CAND1*, the coding region of *CAND1* with the coding sequence of a 3 x FLAG tag (GACTACAAA GACCATGACGGTGATTATAAAGATCATGA CATC GACTACAAGGATGACGATGACAAG) fused to the 3′ end, and the 1 kb genomic DNA sequence immediately downstream of *CAND1*. The expression of the transgenic CAND1 protein was confirmed by analyzing the rosette leaf samples by Western blot, using the HRP conjugated anti-FLAG antibody (Sigma-Aldrich, cat. no. A8592; Supplementary Figure S1C). The genotype of the complementation lines was confirmed by two sets of PCR analyses. The presence of the T-DNA allele was confirmed by PCR using the primers (T and R1) described above (Supplementary Figure S1D). Then, gene specific primers (F: 5′-TATGCTCTTGGAAACATTGCC-3′, and R2: 5′-AAAATTCT TCTTCGCCGATTG-3′) were used to obtain PCR products of the wild-type *CAND1* (from the endogenous loci, or the complementation fragment, or both; Supplementary Figure S1E). These PCR products were digested by HindIII and fractionated on a Novex 4–12% TBE Gel (ThermoFisher Scientific, cat. no. EC62352BOX). Complementation transgenic plants should generate only the DNA fragments of 1.4 kb and 390 bp (Supplementary Figure S1F). Two independent *cand1-3;pCAND1::CAND1^3xFLAG^* complementation transgenic plants (Comp. # 41, # 42) were reported in this study.

### Alexander staining of pollen and anthers

Mature pollen grains or anthers were harvested from freshly opened flowers. To obtain mature pollen grains, anthers were dabbed gently on a microscope slide containing drops of liquid pollen germination medium (0.01% boric acid, 5 mm CaCl_2_, 5 mm KCl, 1 mm MgSO_4_, 10% sucrose, pH 7.5; Boavida and McCormick, 2007). Then, pollen grains were stained in Alexander staining solution (Alexander, 1969). To stain anthers, intact anthers were placed on a microscope slide containing Alexander staining solution (Alexander, 1969) in a humid chamber or following the modified Alexander staining protocol (Peterson et al., 2010). Stained pollen and anthers were observed using a light microscope (Nikon TS2R-C-AL). Each experiment has been performed with at least three biological replicates with similar results.

### Phloroglucinol-HCl staining of anther

To analyze lignin deposition, anthers obtained from freshly opened flowers were stained with 2% (w/v) phloroglucinol-HCl staining solution (made by freshly mixing two volumes of 3% (w/v) phloroglucinol in 95% ethanol with one volume of concentrated HCl; Hao et al., 2014). Stained anthers were observed under a light microscope (Nikon TS2R-C-AL). Each experiment has been performed with at least three biological replicates with similar results.

### DAPI staining of mature pollen

Mature pollen grains were collected from freshly opened Col-0 and *cand1-3* flowers and stained with 0.4 μg/ml DAPI as described previously (Yuan and Kessler, 2019). Stained pollen grains were imaged using a Leica DMI8 fluorescence microscope with a DAPI filter.

### Pollen germination assays

Mature pollen was harvested from freshly opened flowers from Col-0 and *cand1-3* plants. Flowers were washed in 200 μl liquid pollen germination medium in a 1.5 ml centrifuge tube by vortexing for 2 min. Flowers and larger debris were then removed using forceps and the pollen suspension was centrifuged at 13,000–15,000 g for 5 min. The supernatant was discarded, and the pollen pellet was resuspended in 50–250 μl of liquid pollen germination medium. Pollen germination assays were performed at 21°C as previously described (Boavida and McCormick, 2007) and the results were imaged using a Nikon TS2R-C-AL compound microscope.

### Anther sections

The histological analysis was performed as described previously (Zhou et al., 2018; Han et al., 2020a,b). Specifically, closed buds and freshly opened flowers of Col-0 and *cand1-3* plants were fixed in paraformaldehyde (PFA) solution and embedded in Paraplast X (Electron Microscopy Sciences). The paraplast-embedded samples were sectioned with a microtome. The sections were de-waxed, hydrated and stained with Toluidine blue as described previously (Zhou et al., 2015, 2018). Each experiment has been performed with at least three biological replicates with similar results.

### Lignin autofluorescence

The visualization of lignin autofluorescence follows a method described previously (Schuetz et al., 2014; Xue et al., 2020). Anthers were isolated from freshly opened flowers of Col-0 and *cand1-3* mutant, cleared by a clearing reagent (Visikol for Plant Biology) for 15 min, and imaged using a laser scanning confocal microscope (Zeiss LSM 880). The samples were excited at 405 nm and the emission signal was collected from 420 nm to 530 nm. The imaging settings were the same for all anthers analyzed, and the experiment was performed with at least three biological replicates with similar results.

### Analysis of CAND1 expression

Pollen grains of Col-0 and *pCAND1::H2B-GFP* transgenic plants obtained from unopened and freshly opened flowers were imaged using a laser scanning confocal microscope (Zeiss LSM 880) as descried previously (Han et al., 2020b). GFP was excited with a 488-nm laser and the emission signal was collected from 490 nm to 560 nm. The imaging settings were maintained constant for all samples analyzed. Three independent *pCAND1::H2B-GFP* transgenic plants were included in the analysis and similar results were obtained.

### Aniline blue staining of pollen tube in pistil

Pistils 3 days after pollination were dissected and fixed for 4 to 6 hours in the fixing solution (3.7% formaldehyde/formalin, 5% acetic acid, and 50% ethanol), washed twice with water, and incubated overnight in a 4 M sodium hydroxide solution at 4°C. The softened pistils were then washed twice and incubated in de-colorized aniline blue for 2 hours (Huck et al., 2003). The stained samples were imaged using a Leica DMI8 fluorescence microscope with DAPI and Texas Red filters.

## Results

### Expression of F-box protein genes is highly variable among Arabidopsis tissues

**Figure 2:**
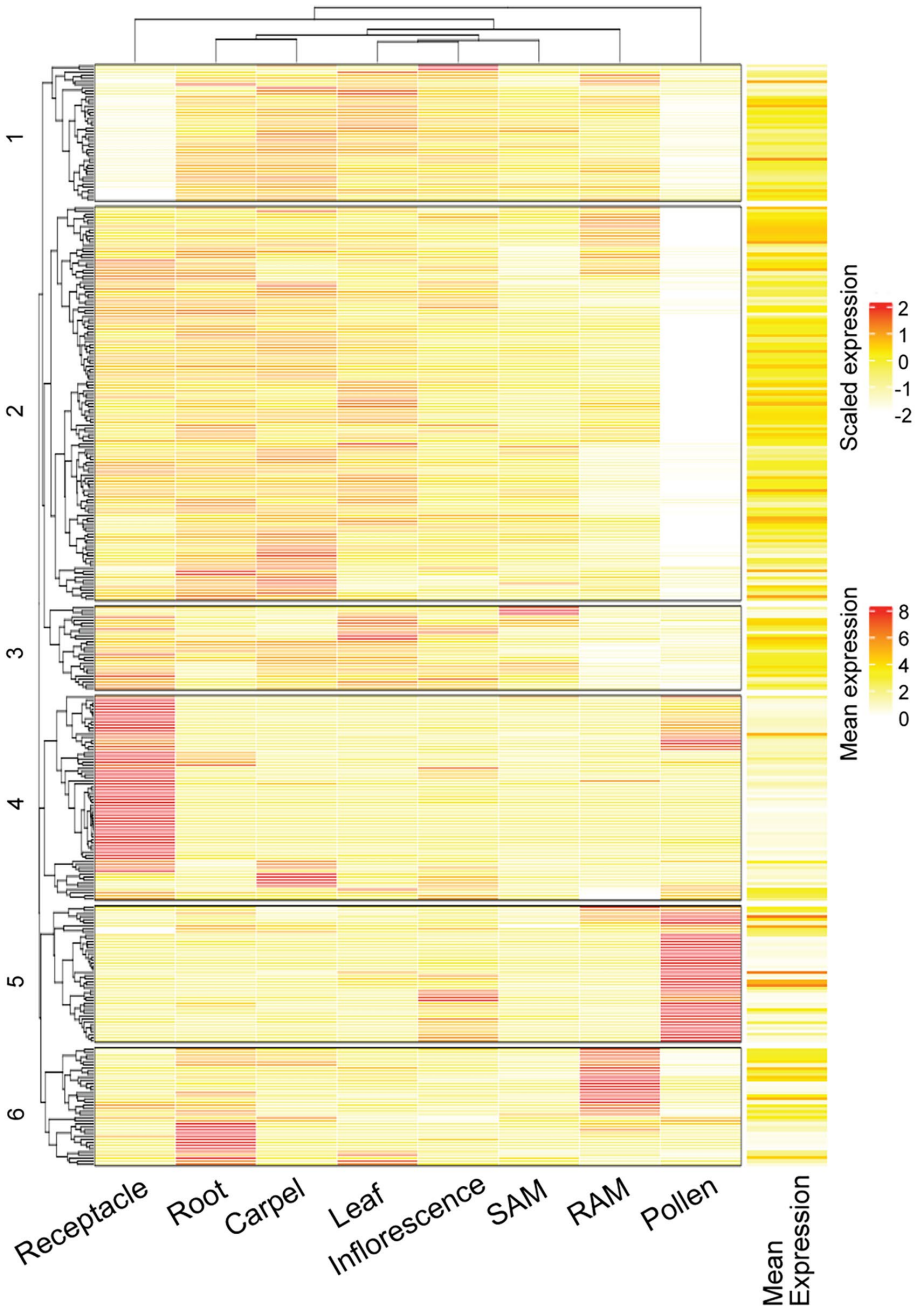
Expression of F-box protein genes are highly variable in different tissues of *Arabidopsis*. Heat map showing RNA-Seq data of 346 F-box protein genes in the indicated tissues of *Arabidopsis*. Both scaled expression (for different tissues) and mean expression are shown. SAM: shoot apical meristem; RAM: root apical meristem. Data are included in Supplementary Table S1.

First, we examined the expression of F-box protein genes in different tissues of *Arabidopsis*. Out of the predicted ~700 F-box protein genes in the Araport database (Krishnakumar et al., 2015; Cheng et al., 2017), we recovered RNA-sequencing data for 468 genes from eight different tissue types (including pollen, receptacle, root apical meristem, stage 12 inflorescence, leaf, shoot apical meristem, root, and carpel). After filtering out low-expression genes, defined as less than 1 Transcript Per Million (TPM) in all the tissues, we generated a heat map for the expression level of 346 F-box protein genes in different tissues (Figure 2; Supplementary Table S1). The heat map indicates that the expression of each F-box protein gene varied significantly in different tissues (shown as rows), and we also found that the type of highly expressed F-box protein genes varied from tissue to tissue as well (see groups 1–6 in Figure 2). For example, the F-box genes in groups 1–3 have no detectable expression in the pollen while groups 4–5 have significantly higher expression. These results suggest that the type of F-box proteins and their level of expression are highly variable during the *Arabidopsis* development and are characteristic of specific cell types and tissues.

### Pollination is impaired in *cand1-3* null mutant

**Figure 3:**
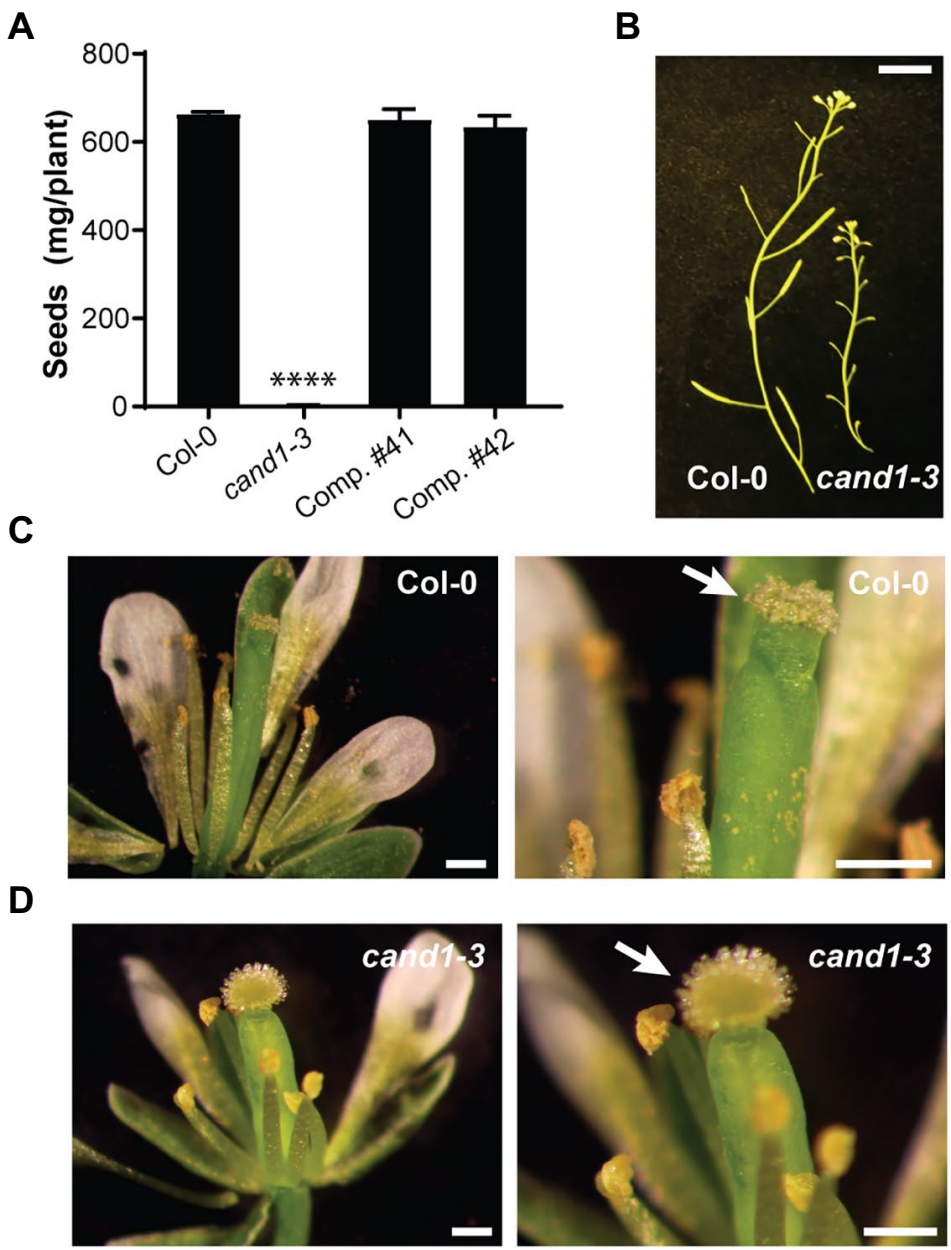
The *cand1-3* mutant displays impaired seed production and poor pollination. **(A)** Weight of seeds collected per wild-type, *cand1-3*, or *cand1-3;pCAND1::CAND1^3xFLAG^* complementation (Comp.) plant. Bars: average weight of seeds collected per plant; error bar: ± standard deviation (SD); *n* = 3; ^****^p  < 0.0001 (two-tailed *t*-test). **(B)** Inflorescence from wild-type and *cand1-3* plants. Scale bar: 1 cm. **(C,D)** Freshly opened flowers from wild-type **(C)** and *cand1-3* **(D)** plants. Close-up views are shown in the right panels, with white arrows pointing to the stigma, which was collapsed in the wild type but projected open in the *cand1-3* mutant plant. Scale bars: 250 μm.

In parallel to the bioinformatic analyses for F-box protein expression, we obtained the *Arabidopsis* T-DNA insertional mutant *cand1-3* (Col-0 background; SALK_110969) that was previously characterized and found to be a recessive null mutant (Cheng et al., 2004; Feng et al., 2004; Alonso-Peral et al., 2006). The *cand1-3*, as well as other *cand1* alleles, display pleiotropic defects such as dwarfism, enhanced shoot branching, and disturbed root growth (Cheng et al., 2004; Chuang et al., 2004; Feng et al., 2004; Zhang et al., 2008). To test the hypothesis that a *cand1* null mutant does not sustain cells which have abundant expression of F-box proteins, we examined phenotypically the *cand1-3* mutant for occurrence of severe cell death in particular cell types or tissues. We noticed that *cand1-3* mutant produced few seeds (Figure 3A), a phenotype previously identified in other *cand1* mutant alleles (Cheng et al., 2004; Feng et al., 2004). The *cand1-3* phenotype was fully complemented when the 12.9-kb genomic fragment including the *CAND1* gene and its regulatory regions was introduced into the *cand1-3* plant (Figure 3A; Supplementary Figure S1). This defect in seed production was not due to impaired development of fruits or flowers in the *cand1-3* mutants: siliques and floral organs such as petals, stamens, and carpels were morphologically similar in both wild-type and *cand1-3* plants (Figures 3B–D). A closer investigation of stigmas in open pollinated flowers revealed that in contrast to the usual collapsed stigmatic papilla of a wild-type flower, the stigmatic papillae of the *cand1-3* self-pollinated flower at an identical developmental stage remained fully expanded (Figures 3C,D, right panels). *Arabidopsis* stigmas are known to collapse after successful pollination (Bosch and Franklin-Tong, 2018; Katano et al., 2020). The absence of collapsed papilla in *cand1-3* thus suggested a defect in pollination.

### The *cand1-3* mutant displays severe defects in pollen production

**Figure 4:**
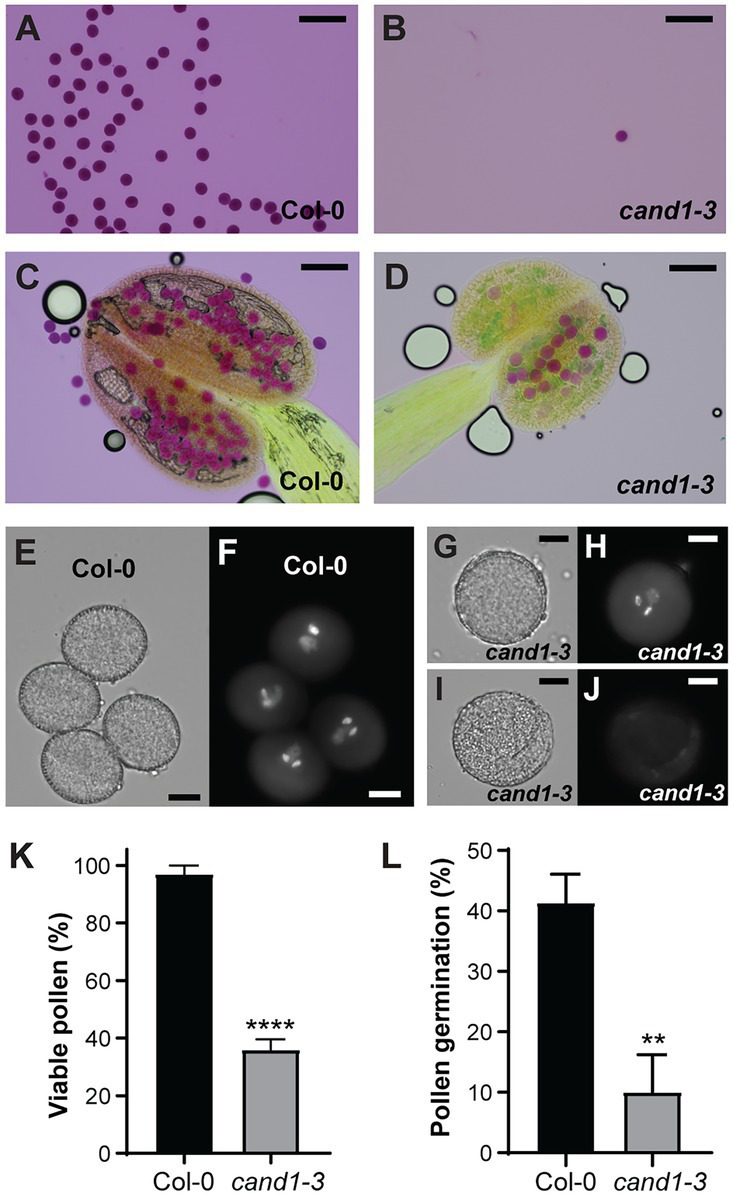
Viability of pollen is severely reduced in *cand1-3* mutant. **(A,B)** Pollen grains obtained from freshly opened flowers of wild-type **(A)** and *cand1-3* **(B)** plants were stained by Alexander staining solution. Scale bars: 100 μm. **(C,D)** Anthers obtained from freshly opened flowers of wild-type **(C)** and *cand1-3* **(D)** plants were stained by Alexander staining solution. Scale bars: 100  μm. **(E–J)** Mature pollen grains obtained from freshly opened flowers of wild-type **(E,F)** and *cand1-3* **(G–J)** plants were stained with DAPI and imaged with a DAPI filter **(F,H,J)** or in transmitted light **(E,G,I)**. The *cand1-3* mutant pollen grains with both positive and negative DAPI stain are shown. Scale bars: 10  μm. **(K)** Quantification of DAPI positive (viable) pollen grains from wild-type and *cand1-3* plants. Bars: average % of viable pollen; error bar: ± SD; *n* = 3 wild-type or mutant plants (30–60 pollen grains per wild-type plant and 10–20 pollen grains per *cand1-3* mutant plant were analyzed); ^****^p  < 0.0001 (two-tailed *t*-test). **(L)** Quantification of the germination rate of pollen grains from wild-type and *cand1-3* plants. Bars: average % germinated pollen; error bar: ± SD; *n* = 3 wild-type or mutant plants (120–400 pollen grains per wild-type plant and 20–120 pollen grains per *cand1-3* plant were analyzed); ^****^p  < 0.0001 (two-tailed *t*-test).

To determine the basis for the poor pollination in *cand1-3* plants, we first compared the number of pollen grains that could be obtained from the wild-type and *cand1-3* mutant open flowers. Mature pollen grains collected from freshly opened flowers were stained with Alexander’s staining solution to reveal pollen developmental defects. While we obtained abundant pollen grains from wild-type plants, pollen grains from *cand1-3* plants were hardly recovered (Figures 4A,B). We then wondered if this difference was because pollen grains were more difficult to extract from *cand1-3* flowers. Therefore, we collected intact anthers from freshly opened flowers and stained them with Alexander’s staining solution. Because Alexander’s solution stains the pollen cytoplasm purple and the pollen wall green, the stain allowed us to distinguish between aborted (lacked cytoplasm) and non-aborted (with cytoplasmic content) pollen grains (Ebel et al., 2004; Tello et al., 2018; Wang and Dobritsa, 2021). We found that most, if not all, wild-type pollen grains stained purple (Figure 4C). In contrast, in the *cand1-3* anther, only few pollen grains stained purple, while the majority was collapsed pollen as identified by the green smear left by the pollen wall structures (Figure 4D). These results suggest that the majority of *cand1-3* pollen grains aborted during pollen development. The pollen abortion phenotype was also fully complemented by the wild-type *CAND1* gene (Supplementary Figure S2A).

To determine if the few *cand1-3* pollen grains that appeared mature and non-aborted had completed development, we used the fluorochrome 4′-6-diamidino-2-phenylindole (DAPI) to stain and visualize the pollen nuclei (Zhu et al., 2014). Almost all pollen from the wild-type flower showed the typical tricellular structure with a less condensed vegetative nucleus and the two condensed sperm cell nuclei (Figures 4E,F). From the few apparently mature pollen grains obtained from *cand1-3* anthers, several presented the typical size, morphology, and cytoplasmic content of a mature pollen grain (Figures 4G,H), suggesting that pollen development was completed. However, a large fraction of these pollen grains had no detectable DAPI stained nuclei (Figures 4I,J). Quantification of DAPI-stained pollen in *cand1-3* showed that the percentage of tricellular pollen was considerably reduced (36%) when compared to the wild type (97%; Figure 4K). These observations suggested that most ca*nd1-3* pollen grains recovered from dehiscent anthers were not viable. In addition, we accessed the ability of *cand1-3* pollen to germinate *in vitro*. Consistent with the above findings, the germination percentage of *cand1-3* pollen was significantly lower than the wild type (Figure 4L). Taken together, these results show that the ability to produce viable pollen is strongly compromised in *cand1-3* mutant plants. These observations also agree with the possibility that nuclear degradation as a result of cell death contributed to the high *cand1-3* pollen lethality.

### The *cand1-3* mutant shows loss of G-type lignin in anther endothecium cells but no defects in anther dehiscence

**Figure 5:**
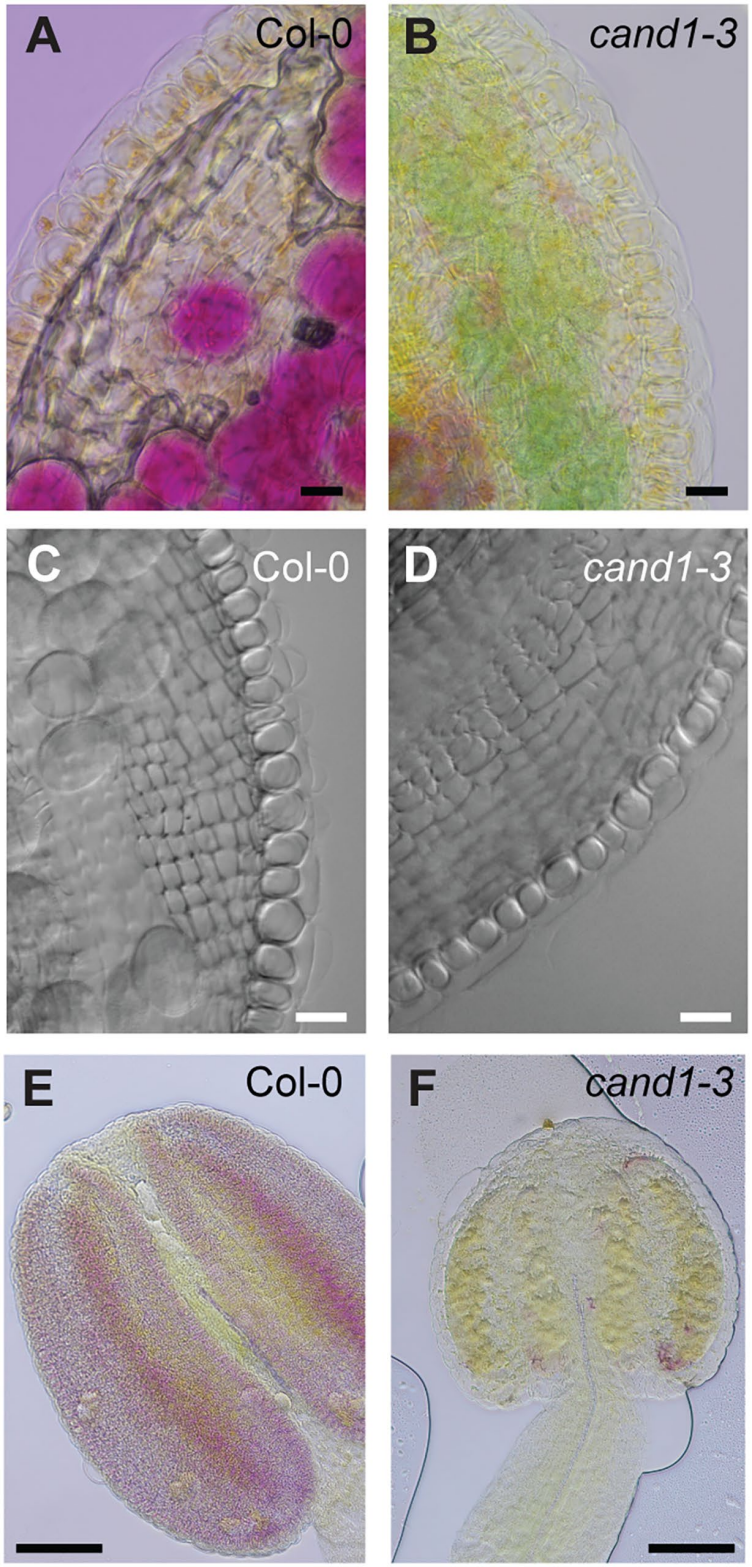
*cand1-3* mutant exhibits reduced lignin content stained by phloroglucinol-HCl. **(A–D)** Both wild-type **(A,C)** and *cand1-3* mutant **(B,D)** anthers contained intact endothecium cell layers. Anthers were stained with Alexander staining solution **(A,B)** or treated with Visikol tissue clearing solution **(C,D)**. Scale bars: 10  μm. **(E,F)** Phloroglucinol-HCl staining of anthers indicates presence of lignin. The wild-type anther was stained red **(E)**, but the *cand1-3* anther showed almost no stain **(F)**. Scale bars: 100  μm.

Defects in anther dehiscence, the programmed rupture of anthers that releases mature pollens, can result in poor pollination and low fertility (Wilson et al., 2011). During this multistage process, the secondary wall thickening of endothecium cells plays a critical role (Wilson et al., 2011). Defects in endothecium cell development or reduced endothecium lignification have been linked with anther indehiscence and male sterility (Yang et al., 2007; Wilson et al., 2011; Fan et al., 2014; Hao et al., 2014; Wang et al., 2015; Dai et al., 2019). Because SCFs have been reported to regulate lignin biosynthesis (Zhang et al., 2015, 2017; Kim et al., 2020) and endothecium secondary wall thickening (Kim et al., 2012), we tested if defective endothecium lignification and anther indehiscence also contributed to the poor pollination observed in *cand1-3* plants. We first analyzed the endothecium cell layer in the wild-type and *cand1-3* anthers and found no major morphological differences (Figures 5A–D). We then used the Wiesner test (phloroglucinol-HCl staining), which detects coniferaldehyde residues in the G lignin (Hao et al., 2014; Blaschek et al., 2020), to visualize lignin deposition in the endothecium. Following the phloroglucinol-HCl staining, the lignified endothecium cells in the wild-type anthers clearly stained red (Figure 5E), while the *cand1-3* anther stain was significantly weaker (Figure 5F). Again, this phenotype is absent in the anthers from the *CAND1* complementation plants (Supplementary Figure S2B).

**Figure 6:**
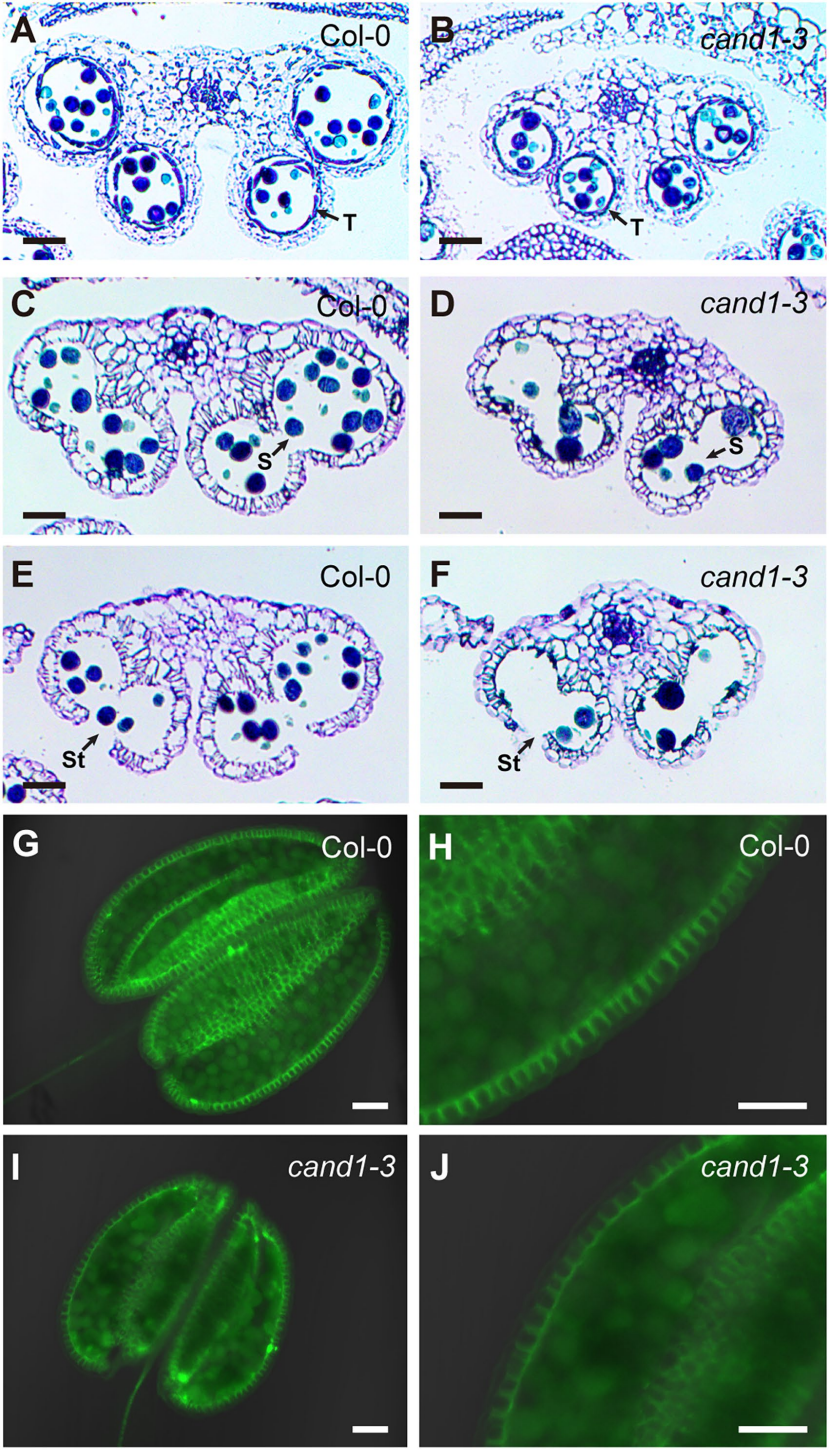
Loss of *CAND1* leads to pollen abortion but does not affect anther dehiscence. **(A,B)** Images of transverse sections of wild-type **(A)** and *cand1-3* mutant **(B)** anthers from unopened flowers, representing developmental stage 11. Arrows point to tapetum (T). **(C–F)** Images of transverse sections of wild-type **(C,E)** and *cand1-3* mutant **(D,F)** anthers from freshly opened flowers. Wild-type **(C)** and *cand1-3* mutant **(D)** anthers exhibited open septum (S, as pointed in the figure), representing developmental stage 12. Wild-type **(E)** and *cand1-3* mutant **(F)** anthers exhibited open septum and stomium (St, as pointed in the figure), representing developmental stage 13. **(G–J)** Lignin autofluorescence in the endothecium layer of the wild-type **(G,H)** and *cand1-3* mutant **(I,J)** anther. Scale bars: 50  μm.

The lack of positive phloroglucinol-HCl staining often suggests the lack of lignin deposition in anther endothecium, which has been previously reported to affect anther dehiscence (Fan et al., 2014; Hao et al., 2014; Wang et al., 2015; Dai et al., 2019). To investigate if the *cand1-3* anther failed to dehisce properly, we collected anthers at different developmental stages (stage 11–13; Sanders et al., 1999) and analyzed their cell anatomy. At stage 11, the *cand1-3* anthers showed well-defined cell layers including the presence of densely stained layer of tapetum cells similar to the wild type (Figures 6A,B). As expected, the anther septum and stomium remained closed (Sanders et al., 1999; Figures 6A,B). At stage 12, the septum in both the wild-type and *cand1-3* anthers was fully open (Figures 6C,D), and at stage 13, an open stomium was visible in both backgrounds (Figures 6E,F). These results suggest that the low fertility in *cand1-3* was not caused by defects in anther dehiscence. Further, they suggest that the lack of positive stain in *cand1-3* anthers in the Wiesner test is likely due to changes in lignin composition instead of reduced lignin deposition in the endothecium cell wall (Blaschek et al., 2020). To test this possibility, we compared lignin autofluorescence as a measure of total lignin content in wild type and *cand1-3* anthers (Xu et al., 2019; Donaldson, 2020; Xue et al., 2020). In contrast to the strong difference revealed by the Wiesner test (Figures 5E,F), the autofluorescent signal from *cand1-3* anthers did not differ much from that in the wild-type anthers (Figures 6G–J). This result suggests that the lignin composition, rather than the level of total lignin, was significantly changed in the *cand1-3* anther endothecium.

### Pollen expresses the highest level of F-box proteins

**Figure 7:**
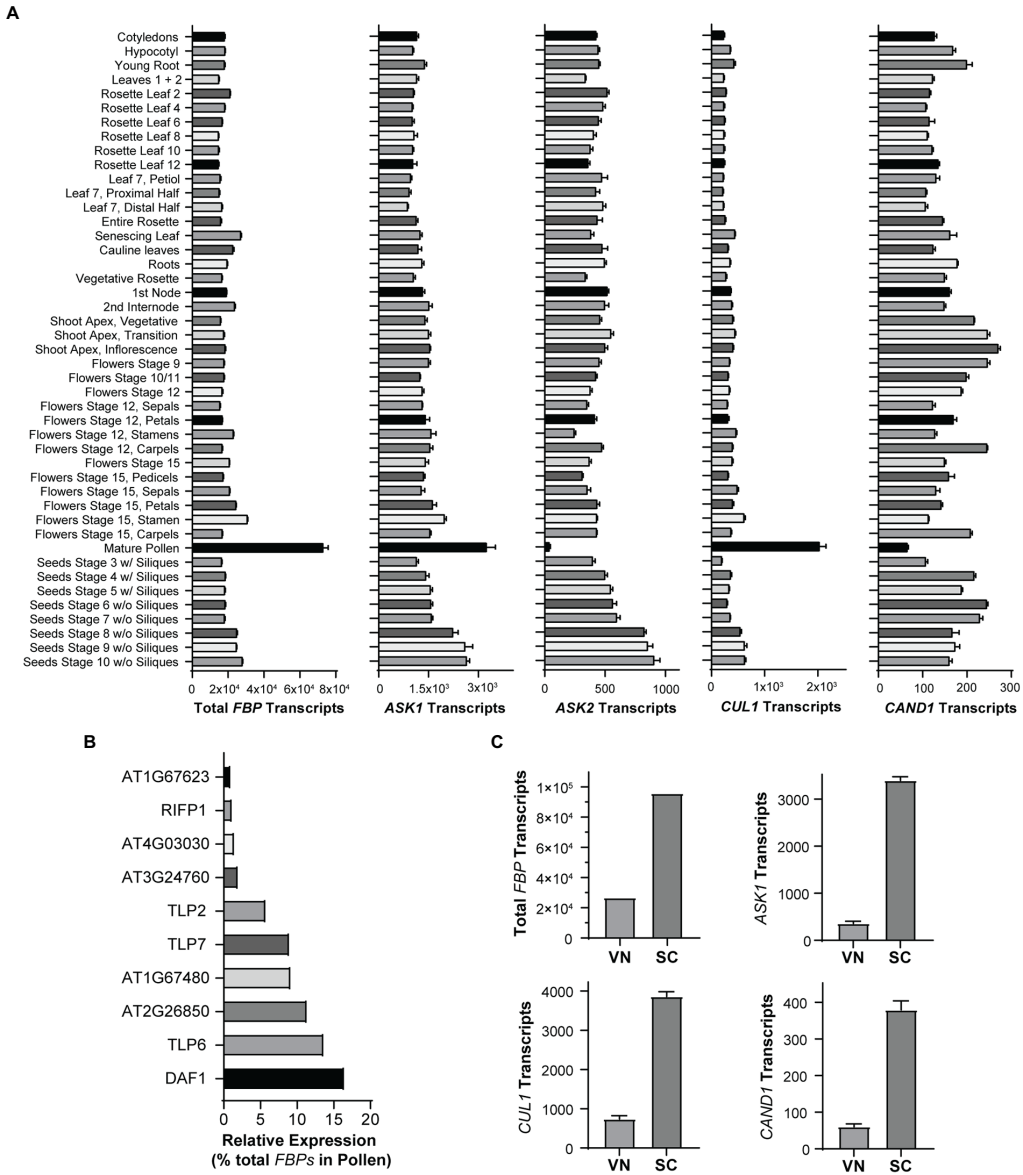
Transcription of F-box protein genes is highly active in mature pollen. **(A)** Expression levels of total F-box protein (*FBP*), *ASK1*, *ASK2*, *CUL1*, and *CAND1*, in the indicated tissues of *Arabidopsis*. Quantitative gene expression data from 45 tissue types were obtained from ePlant. Expression levels of 406 F-box protein genes were summed and plotted in the graph as total *FBP* transcripts. Error bars: ± standard error; *n* = 3. See also Supplementary Tables S3, S4. **(B)** F-box protein genes highly expressed in pollen. Genes with relative expression level ≥ 1% of the total *FBP* transcripts in **(A)** are shown. **(C)** Expression levels of total *FBP*, *ASK1*, *CUL1*, and *CAND1* in vegetative nuclei (VN) and sperm cells (SC). See also Supplementary Table S5.

Our findings that *cand1-3* plants develop anthers that have normal structures and correctly undergo programmed dehiscence support our interpretation that cell death during pollen development is the major cause for *cand1-3* poor pollination and low fertility. In fact, pollen cell death was increasingly severe during pollen development in *cand1-3*. While comparable numbers of pollen grains were observed in stage 11 anthers of wild type and *cand1-3* mutant (Figures 6A,B), a significant reduction on the number of pollen grains was observed in stage 12 and stage 13 *cand1-3* anthers (Figures 6C–F), explaining the small number of viable pollen grains obtained from *cand1-3* anthers (Figure 4). Therefore, pollen is the tissue that cannot be maintained when CAND1 is lost. To further analyze if this finding is consistent with the adaptive exchange hypothesis (Figure 1), we estimated the size of the total F-box protein pool in different *Arabidopsis* tissues. First, we summed up levels of F-box protein transcripts in individual tissue types (Figure 2) and found that pollen contains the largest total pool of F-box protein transcripts among the eight plant tissues analyzed (Supplementary Figure S3). To extend our analysis to a more comprehensive set of tissues and developmental stages, we compiled quantitative gene expression data from the ePlant database (Waese et al., 2017). In ePlant, we gathered triplicated values for expression levels of 406 F-box protein genes that were normalized across 45 tissue types (Schmid et al., 2005; Waese et al., 2017). The gene expression trend in similar tissues from the ePlant and Araport datasets generally align with each other (Supplementary Table S2). After summing up the levels of F-box protein transcripts from each tissue type, we confirmed that mature pollen contains a total pool of F-box protein transcripts significantly larger than any other tissue included in this dataset (Figure 7A; Supplementary Table S3). This result is therefore consistent with the adaptive exchange hypothesis, which states that the CAND1-mediated exchange of F-box proteins enables cells to tolerate large variations in the expression of F-box protein genes. The identity of F-box protein genes highly expressed in pollen are shown in Figure 7B.

Next, we extended our analysis to *Arabidopsis-SKP1-like* (*ASK*) genes (Supplementary Table S4) that produce the adaptor proteins necessary for docking F-box proteins on CUL1. Interestingly, *ASK1*, the major and most highly expressed *ASK* gene, has the highest expression level in pollen, while *ASK2*, the second most highly expressed *ASK*, expresses at the lowest level in pollen (Figure 7A). We also graphed the expression level of *CUL1* and *CAND1* in different tissues (Figure 7A). As the mature male gametophyte is a multicellular structure consisting of two cell types that differ significantly in their transcriptional program and cell fate, we analyzed the distribution of gene transcripts in the pollen vegetative nuclei (VN) and sperm cells (SC; Supplementary Table S5). Using cell type specific datasets generated in previous studies (Jiang et al., 2015; Borg et al., 2020), we found that the total transcripts of F-box protein genes, and the transcripts of *ASK1*, *CUL1*, and *CAND1*, are primarily localized in sperm cells (Figure 7C).

**Figure 8:**
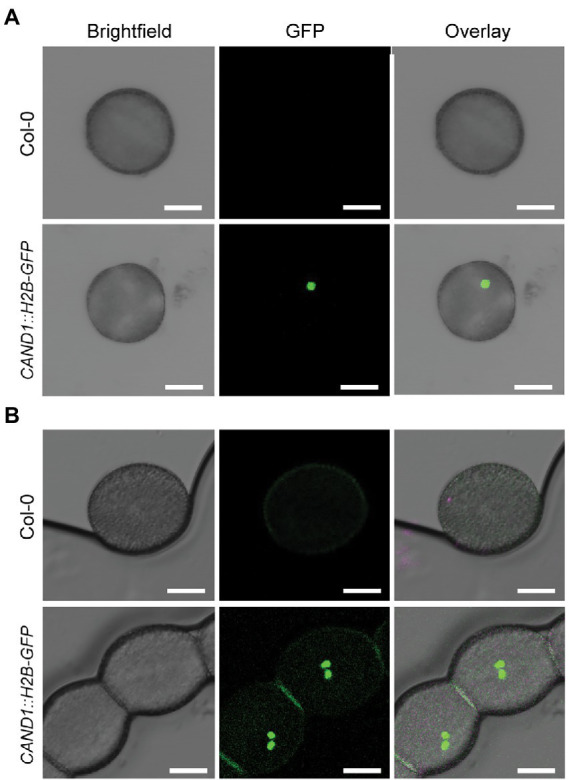
Expression of *CAND1* during pollen development. **(A)** Images of bicellular pollen grains obtained from wild-type and *pCAND1::H2B-GFP* transgenic plants. Brightfield, GFP, and overlay (merged brightfield and GFP) images are shown. **(B)** Images of tricellular/mature pollen grains obtained from wild-type and *pCAND1::H2B-GFP* transgenic plants. Brightfield, GFP, and overlay (merged brightfield and GFP) images are shown. Scale bars: 10 μm.

To examine *CAND1* expression in pollen, we transformed wild-type *Arabidopsis* with the coding sequence of Histone 2B-GFP (H2B-GFP; Han et al., 2020b) driven by the *CAND1* promoter (*pCAND1::H2B-GFP*). Confocal microscopy validated that *CAND1* was expressed in the generative cell of bicellular pollen (Figure 8A) and in the two sperm cells of mature tricellular pollen (Figure 8B; Saleme et al., 2021). The lack of detectable GFP signal in vegetative nuclei suggested that *CAND1* was expressed at significantly lower levels in the pollen vegetative cell, which is consistent with the transcriptomic data (Figure 7C). All together, these results support the conclusion that CAND1, the F-box protein exchange factor, is essential for viability of tissues or cell types that express high levels of F-box proteins.

### *cand1-3*/+ heterozygous plants have reduced male gametophytic transmission

**Figure 9:**
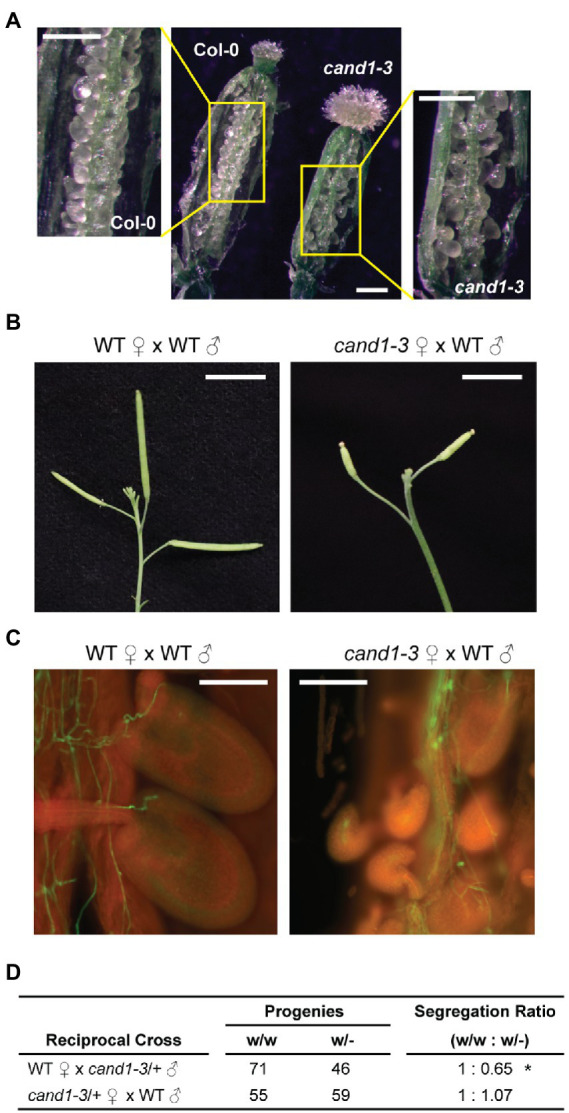
Reduced male gametophytic transmission in *cand1-3*/+ heterozygous plants. **(A)** Ovules in ovaries of wild-type and *cand1-3* plants. Scale bars: 250  μm. **(B)** Siliques of the wild-type or *cand1-3* plant 7  days after pollination with wild-type pollen grains. Scale bars: 1 cm. (C) Aniline blue staining of wild-type pollen tubes growing in wild-type or *cand1-3* pistils 3 days after pollination. Scale bars: 150 μm. (D) Segregation ratio after reciprocal crosses of wild-type and *cand1-3*/+ heterozygous plants. ^*^p  < 0.05 (chi-square goodness of fit test).

Though the severe defect in pollen viability alone is sufficient to cause poor fertility, defects in ovule development may also contribute to the low seed yield in *cand1-3* plants. We therefore carefully examined the ovary of the wild-type and *cand1-3* plants. Despite a smaller size, the ovary of the *cand1-3* plant displayed correct structures and produced a good number of ovules (Figure 9A). We pollinated the *cand1-3* stigma with wild-type pollen and performed a control cross with wild-type plants to assess if the *cand1-3* ovules were fully functional and could produce seeds. Siliques from hand-pollinated pistils were examined 7 days after pollination. The *cand1-3* mutant failed to develop elongated siliques (Figure 9B) and yielded no seeds. Although wild-type pollen successfully geminated and grew in *cand1-3* pistils, many of the pollen tubes failed to target the ovules (Figure 9C). These results suggest that in addition to the observed defects in the male gametophyte, the female gametophyte in *cand1-3* homozygous plants was also defective.

We then wonder if the loss of *CAND1* affects the development of male and/or female gametophytes, independent of the pleiotropic defects in the parental *cand1-3* homozygous plants. Thus, we performed reciprocal crosses between wild-type and *cand1-3*/+ heterozygous plants and examined the genotypes of the resulting progeny. If *cand1-3* male or female gametophytes are defective, we would expect distortion from the normal segregation ratio (wild type: heterozygote = 50%: 50%). Indeed, we observed a 35% reduction in *cand1-3* transmission efficiency when *cand1-3*/+ pollen was used to pollinate a female wild-type pistil (Figure 9D). These results are consistent with defects affecting the male, but not the female gametophyte.

## Discussion

How CAND1 regulates SCFs had been a puzzling question for many years until the dynamic interactions among CUL1, CAND1, and F-box proteins were evaluated in a kinetic study. The study showed that the spontaneous dissociation of an SCF complex is extremely slow, but in the presence of CAND1, the existing SCF complex can be quickly disassembled and the CUL1 can be accessed by another F-box protein to assemble a new SCF (Pierce et al., 2013). This finding revealed a fundamental mechanism for the current model of CUL1 cycling (Figure 1 and introduction), and it suggested that CAND1 is a key factor for SCF activation. Consistent with conclusions from the kinetic study, the SCF substrate protein was degraded more slowly in human HEK293 cells lacking the *CAND1* and *CAND2* genes (DKO cells; Liu et al., 2018). However, the DKO cells displayed no apparent defects in morphology or cell division rates, suggesting that at least under the cell culture condition, cells can develop ways to keep the SCF system functional without CAND1 (Liu et al., 2018). In addition, when the level of CUL1 was elevated in the DKO cells, most F-box proteins were assembled with CUL1, and the defect of SCF-catalyzed protein degradation was mitigated (Liu et al., 2018). This finding suggests that if cells produce CUL1 at a level saturating the SCF assembly, an exchange mechanism becomes dispensable, which begs the question: why does the exchange system emerge and remain evolutionarily conserved?

The adaptive exchange hypothesis was proposed to explain the necessity of the CAND1-mediated exchange system. This hypothesis suggests that through rapidly exchanging the F-box proteins in the SCF complexes, most, if not all, F-box proteins can access CUL1 to form active SCFs. As a result, cells remain fully functional even when large variations in F-box protein expression occur due to changes in developmental programing or environmental condition. In the absence of the adaptive exchange, cells would have to establish machineries to ensure that levels of CUL1 were sufficient to saturate all available F-box proteins under any conditions. This idea was supported by computational simulation and experiments using human HEK293 cells overexpressing F-box proteins (Liu et al., 2018), and here we initiated this study to test the hypothesis using a multicellular organism without introducing gene overexpression.

The flowering plant *A. thaliana* expresses hundreds of F-box proteins. The level and type of expressed F-box proteins vary substantially from tissue to tissue, suggesting that the expression of F-box protein genes is dynamically regulated during *Arabidopsis* development. Based on the adaptive exchange hypothesis, we predicted that tissues or cell types that express the highest level of F-box proteins may display the most severe defects when *CAND1* is lost. Consistent with this prediction, we found that the *cand1-3* homozygous plants fail to produce viable pollen grains, a multicellular structure that express the highest level of total F-box proteins during the development of *Arabidopsis*. Furthermore, the transmission efficiency of the *cand1-3* allele was reduced through male but not female gametophytes, demonstrating that the male gametophyte is the most affected tissue in the absence of CAND1. Altogether, these findings provide further support for the adaptive exchange hypothesis.

Among the top ten highly expressed F-box protein genes in pollen (Figure 7B), three are from the Tubby-like protein (TLP) family (*TLP6*, *TLP7*, *TLP2*). Consistently, defects in male gametophyte development were previously reported in their loss-of-function mutants (Renak et al., 2012). The transcription of *ASK1* also peaks in pollen, supporting the interpretation that F-box proteins capable of assembling SCFs are highly abundant in pollen. The high level of *ASK1* and low level of *ASK2* in pollen echo the previous understanding of conserved and divergent roles of these two genes in male gametophyte development (Zhao et al., 2003). The *CAND1* expression level also varies, and the level in pollen is ~25% of that in inflorescence. This range of variation may not impact SCF activity, because previous data have shown that SCF substrate stability remained unchanged when CAND1 concentration was experimentally reduced to 13% of the normal level, or was mathematically varied across a broad range of concentrations in computational modeling (Liu et al., 2018). Interestingly, pollen expresses *CUL1* at a level significantly higher than any other tissue. This is unexpected because an anticipated advantage of the CAND1-mediated exchange system is that cells can adapt to variable expression levels of F-box proteins while maintaining a relatively steady CUL1 pool. Previous attempts to overexpress CUL1 in *Arabidopsis* (Col-0) failed to recover transgenic overexpression lines, suggesting that an overall increase of CUL1 expression is deleterious to plants (Hellmann et al., 2003). The increased expression of both F-box protein and *CUL1*, and the decreased expression of *CAND1* in pollen point to the possibility that pollen requires higher concentrations of SCF E3 ligases for optimal rates of protein ubiquitination. This may be because SCF substrates in pollen are present at high concentrations, the substrates bind the corresponding SCFs with low affinities, or the substrates have to be ubiquitinated in a short time frame. Further studies on the global SCF assembly and the ubiquitinated proteome will help explain the observed gene expression pattern in pollen.

In this study, we used gene transcript levels as an estimate of protein abundance. A major caveat of this experimental approach is that steady-state mRNA levels do not always correlate to abundance of proteins. We chose this approach because: (1) the publicly available transcriptomic datasets have covered a large variety of tissues and developmental stages; (2) the lower detection limit for gene transcripts makes quantitative gene expression measurements more feasible using limited sample material such as *Arabidopsis* pollen; and (3) quantitative proteomic datasets are still limited for specific tissues or conditions. Nevertheless, we retrieved quantitative proteomic data (Mergner et al., 2020) and analyzed the 160 F-box proteins listed in at least one of the 26 *Arabidopsis* tissues. The protein abundance of total F-box proteins, ASK1, ASK2, CUL1, and CAND1 are summarized in Supplementary Figure S4; Supplementary Table S6, showing no substantial variation across different tissues. Further efforts are needed to define the absolute concentrations, working mechanisms, and biological roles of F-box proteins in different tissues.

Our work confirms that CAND1, an activator for SCF ubiquitin ligases, plays an essential role in pollen development. Through targeting protein substrates for ubiquitination, SCFs modulate the stability and function of many regulatory proteins that play crucial roles in cells (Hua and Vierstra, 2011). While transcriptomic and genetic studies have identified F-box protein genes strictly regulated during pollen development (Honys and Twell, 2004; Borges et al., 2008), further biochemical, genetic, and physiological studies are needed to define the CUL1-dependent (and probably also CUL1-independent) functional mechanism for each of these F-box proteins. Anther and pollen development and the timely release of pollen grains have significant importance in crop breeding (Gomez et al., 2015), where manipulation of male fertility is a desired trait for hybrid seed production (Singh et al., 2015). Therefore, understanding the role of CAND1 and SCFs in the underlying mechanism essential to normal pollen development is of considerable interest.

We found that the lignin composition, rather than total lignin deposition, was changed in *cand1-3* anthers. The reduction in coniferaldehyde content could be due to increased activity of cinnamyl-alcohol dehydrogenase (CAD) that converts coniferaldehyde to coniferyl alcohol, or increased activity of ferulate 5-hydroxylase (F5H) that converts coniferaldehyde to 5-hydroxyconiferaldehyde which eventually forms S lignin (Vanholme et al., 2019). Because modifying lignin composition is of special interest in improving biofuel production (Lockhart, 2015; Muro-Villanueva et al., 2019), further studies into the regulatory role of SCFs in lignin biosynthesis may provide new lines of research in the field.

## Data availability statement

The datasets presented in this study can be found in online repositories. The names of the repository/repositories and accession number(s) can be found in the article/Supplementary material.

## Author contributions

LCB, YZ and XL contributed to the conception and design of the study. LL, MG, JY, and SHL performed the experiments and data analyses. LCB assisted with experimental procedures and data analysis. XW and KV analyzed transcriptomic and proteomic datasets. All authors discussed and commented on the research results. MG wrote the initial manuscript. KV, LCB, YZ and XL substantively revised the initial manuscript. All authors contributed to the manuscript revision, read, and approved the manuscript.

## Funding

This work was supported by National Institutes of Health grant R35 GM138016 (to XL), R01 GM143268 (to YZ), and Hatch fund from USDA-NIFA (to XL). This work was also supported by a Bird Stair Graduate Fellowship (to MG).

## Conflict of interest

The authors declare that the research was conducted in the absence of any commercial or financial relationships that could be construed as a potential conflict of interest.

## Publisher’s note

All claims expressed in this article are solely those of the authors and do not necessarily represent those of their affiliated organizations, or those of the publisher, the editors and the reviewers. Any product that may be evaluated in this article, or claim that may be made by its manufacturer, is not guaranteed or endorsed by the publisher.
