# Supplementary material for: CAND1 is required for pollen viability in Arabidopsis thaliana—a test of the adaptive exchange hypothesis
Source: Front Plant Sci. 2022 Jul 28;13:866086. doi: 10.3389/fpls.2022.866086 (PMC9366119; doi:10.3389/fpls.2022.866086)
Supplement: Supplementary file 2 [file Data_Sheet_1.PDF]

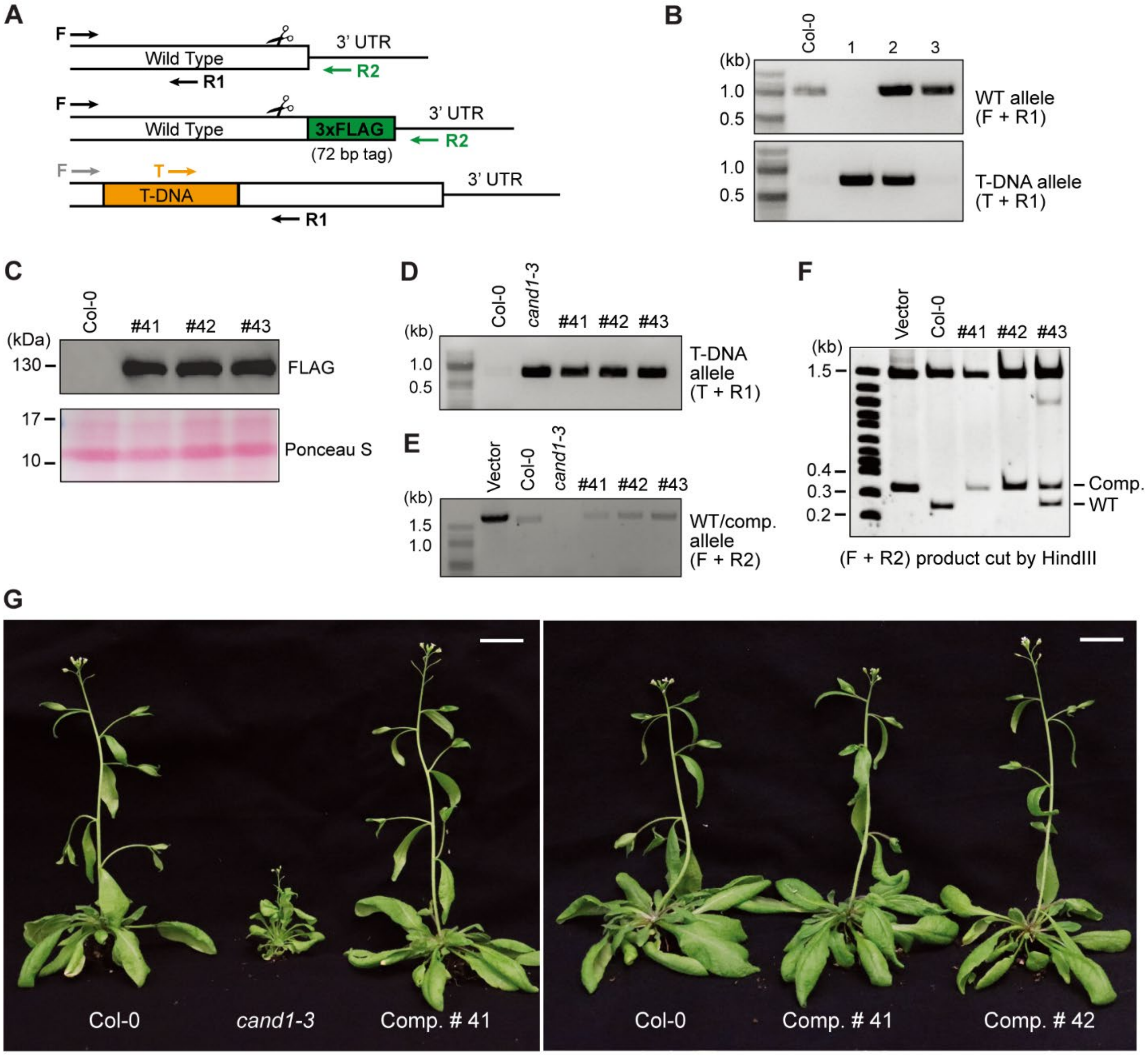


**Supplementary Figure S1. Expression of *pCAND1::CAND1*$^{3xFLAG}$ fully complements the phenotypic defects of homozygous *cand1-3* plants (related to Figure 3). (A)** Schematic illustrating primers used for genotyping (see also Experimental procedures). Primer set of F and R1 detect the wild-type *CAND1* gene; T and R1 identify the T-DNA insertion; F and R2 detect the exogenous *CAND1*$^{3xFLAG}$ gene, which is 72 bp longer than the endogenous copy, if any. **(B)** Images of representative genotyping results for progenies of *cand1-3*/+ heterozygous plants. Col-0: genomic DNA from a Col-0 plant was used for the genotyping PCR to serve as a wild-type control. Sample 1: representative result for a *cand1-3* homozygous plant. Sample 2: representative result for a *cand1-3*/+ heterozygous plant. Sample 3: representative result for a wild-type plant. **(C-F)** Genotypic confirmation of the *cand1-3;pCAND1::CAND1*$^{3xFLAG}$ complementation plants. **(C)** The CAND1$^{3xFLAG}$ protein was detected in the transgenic plants via Western-blot using HRP conjugated anti-FLAG antibody. Col-0 was included as a negative control. Results for three independent transgenic plants, named #41, #42, and #43, are shown. Ponceau S stain served as a loading control. **(D)** The presence of the *cand1-3* T-DNA was confirmed in transgenic lines #41-43, using primers T and R1. Col-0 and *cand1-3* were included as negative and positive controls, respectively. **(E)** The presence of the

complementation and/or the wild-type *CAND1* gene was confirmed in transgenic lines #41-43, using primers F and R2. The *pCAND1::CAND1*$^{3xFLAG}$ plasmid (Vector) and Col-0 served as positive controls, and *cand1-3* served as a negative control. **(F)** PCR products in (**E**) were digested by HindIII and fractionated on a 4-12% TBE gel. The presence of a DNA band below the 0.3 kb maker demonstrated the presence of the endogenous wild-type *CAND1* gene. Results of (**C-F**) confirmed that the transgenic plants #41 and #42 were the *cand1-3;pCAND1::CAND1*$^{3xFLAG}$ complementation lines. **(G)** The complementation lines #41 and #42 are phenotypically similar to the wild-type plants. Scale bars: 2 cm.

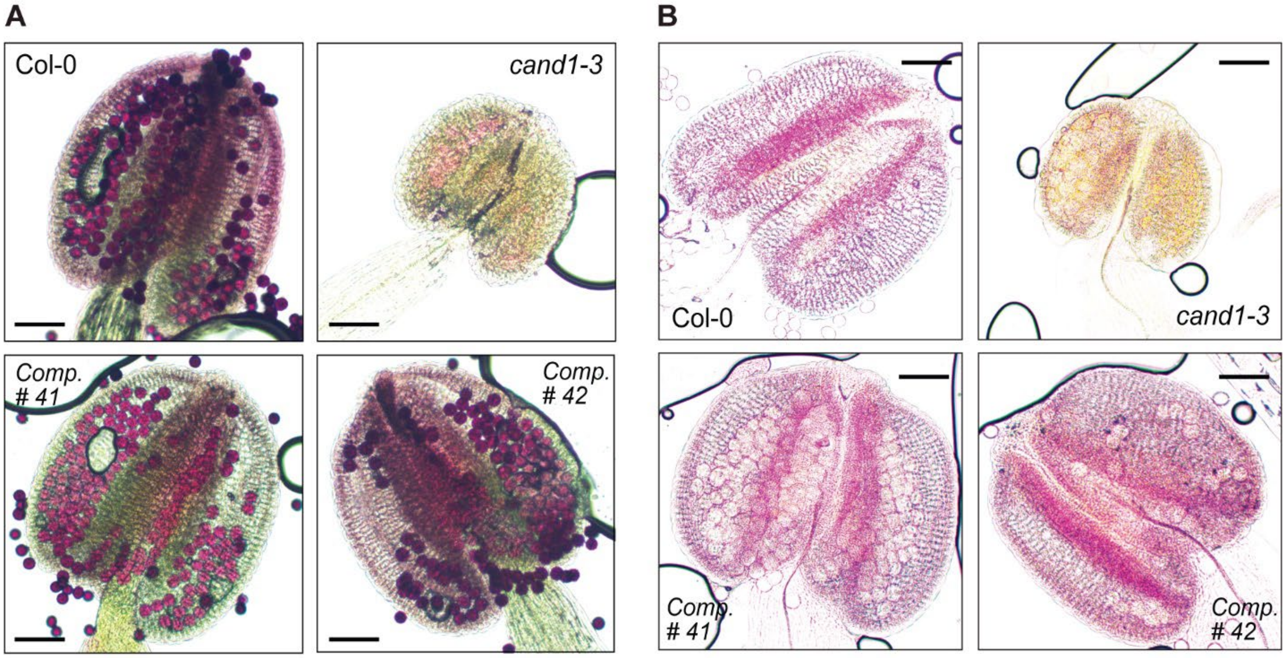


**Supplementary Figure S2. Complementation of defects in pollen viability and G lignin deposition in *cand1-3* (related to Figure 4 and Figure 5). (A)** Anthers obtained from freshly opened flowers of the indicated plants were stained by Alexander staining solution. Scale bars: 100 µm. **(B)** Phloroglucinol-HCl staining of anthers from indicated plants. Scale bars: 100 µm.

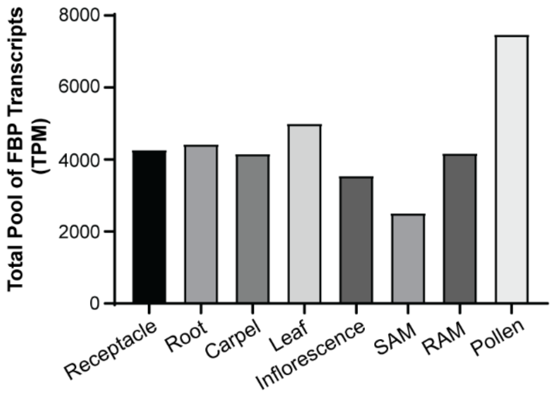


**Supplementary Figure S3. Total pool of F-box protein (FBP) transcripts in the indicated tissues of *Arabidopsis* (related to Figure 2 and Figure 7).** RNA-Seq data of the 346 F-box protein genes from each tissue as in Figure 2 were summed and plotted in the graph.

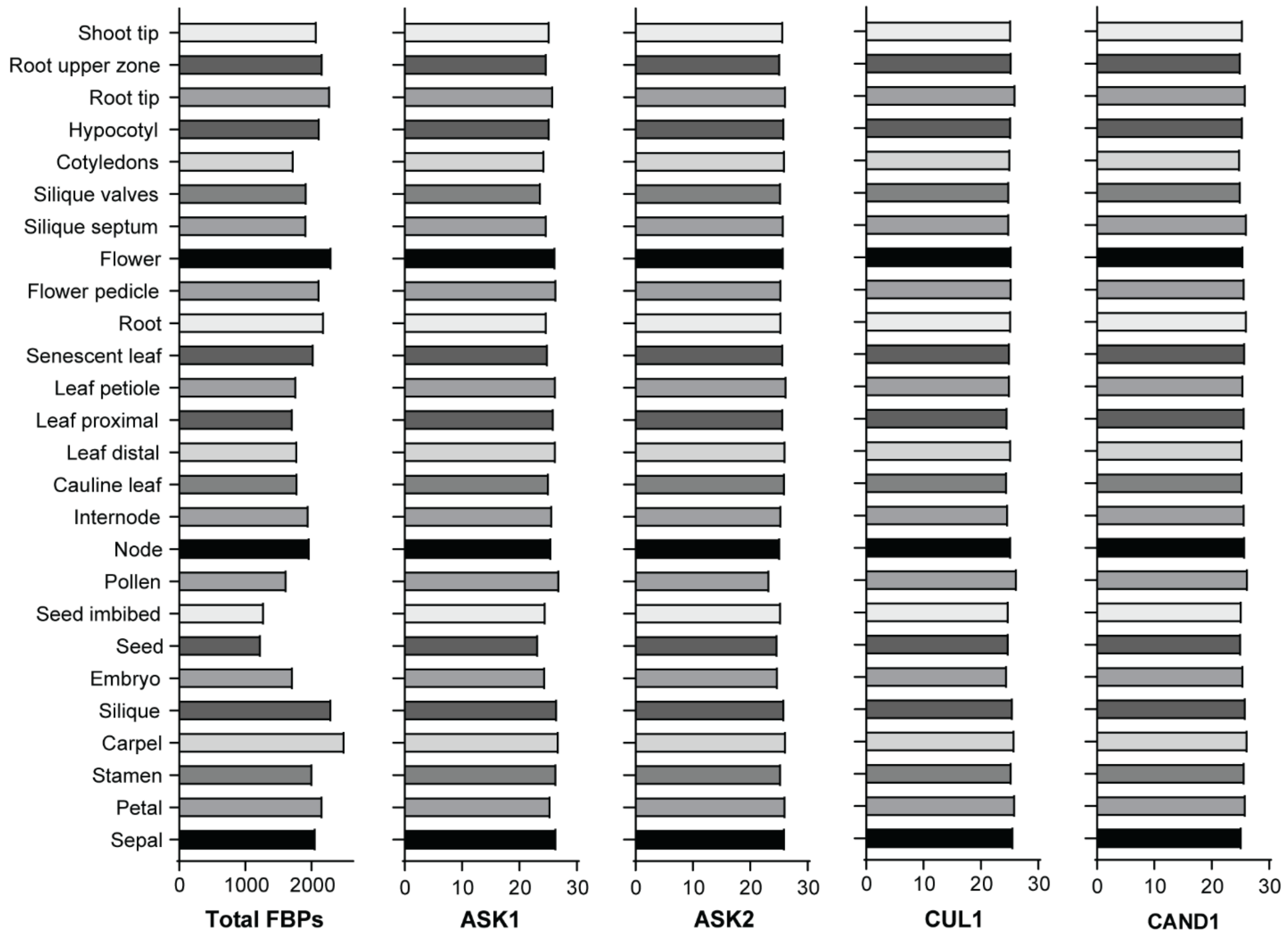


**Supplementary Figure S4. Proteomic data for total F-box proteins (FBPs), ASK1, ASK2, CUL1, and CAND1 in different *Arabidopsis* tissues.** Variation of protein abundance across different tissues for total FBPs: ± 0-36% from the mean; ASK1: ± 0-7% from the mean; ASK2: ± 1-9% from the mean; CUL1 and CAND1: ± 0-3% from the mean. Data are included in Supplementary Table S6.

**Supplementary Table S1. RNA-Seq data for 346 F-box protein genes in eight different *Arabidopsis* tissues** (Araport)

See Excel file

**Supplementary Table S2. Transcriptomic datasets of similar tissues retrieved from Arapot and ePlant**

See Excel file

**Supplementary Table S3. Quantitative transcriptomic data for F-box protein genes in different *Arabidopsis* tissues** (ePlant)

See Excel file

**Supplementary Table S4. Quantitative transcriptomic data for *ASK* genes in different *Arabidopsis* tissues** (ePlant)

See Excel file

**Supplementary Table S5. Quantitative transcriptomic data for F-box protein genes, *ASK1*, *CUL1*, *CAND1* in *Arabidopsis* pollen vegetative nuclei (VN) and sperm cells (SC)** (Jiang et al., 2015; Borg et al., 2020)

See Excel file

**Supplementary Table S6. Quantitative proteomic data for 160 F-box proteins, ASK1, CUL1, and CAND1 in different *Arabidopsis* tissues** (Mergner et al., 2020)

See Excel file
